# Supplementary figures and images for: Effects of pre-transport diet, transport duration and transport condition on immune cell subsets, haptoglobin, cortisol and bilirubin in young veal calves
Source: PLoS One. 2021 Feb 16;16(2):e0246959. doi: 10.1371/journal.pone.0246959 (PMC7886138; doi:10.1371/journal.pone.0246959)

A)

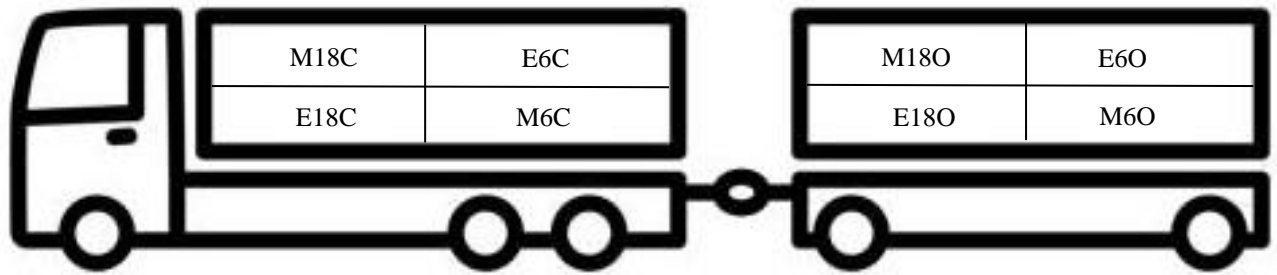

B)

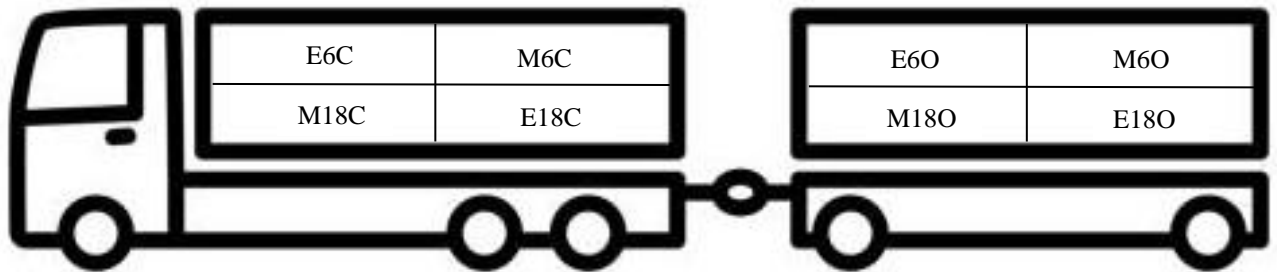

Supplement: S1 Fig — Design of the truck and the trailer for batch 1 (A) and batch 2 (B) of calves. M6C = milk, 6 hours transport, conditioned truck; M18C = milk, 18 hours transport, conditioned truck; M6O = milk, 6 hours transport, open truck; M18O = milk, 18 hours transport, open truck; E6C = electrolytes, 6 hours transport, conditioned truck; E18C = electrolytes, 18 hours transport, conditioned truck; E6O = electrolytes, 6 hours transport, open truck; E18O = electrolytes, 18 hours transport, open truck. (PDF) [file pone.0246959.s001.pdf]
