# Supplementary material for: Effects of pre-transport diet, transport duration and transport condition on immune cell subsets, haptoglobin, cortisol and bilirubin in young veal calves
Source: PLoS One. 2021 Feb 16;16(2):e0246959. doi: 10.1371/journal.pone.0246959 (PMC7886138; doi:10.1371/journal.pone.0246959)
Supplement: S1 Table — 1% = proportion relative to lymphocytes; 2SEM = standard error of the means; 3Perf+ = CD8+ T cells, NK cells, CD4+ and δγ+ T cells were stimulated with perforin to examine the functionality of these cells and how their functionality was affected by the different treatments. (PDF) [file pone.0246959.s002.pdf]

S1 Table. Effects of diet composition (electrolytes vs milk) at the collection centre (CC), transport condition (conditioned vs open truck) and transport duration (6 vs 18 hours) on the difference between CC and post-transport (T0) measurements (deltas, Δ = T0 – CC) of different cell subsets in blood of young veal calves (LS means).

	Pre-transport diet				Transport condition				Transport duration			
Parameter (%)[1]	Electrolytes	Milk	SEM[2]	*P*-value	Conditioned	Open	SEM	*P*-Value	6 h	18 h	SEM	*P*-Value
CD8+ T cells	-0.67	-1.26	0.25	0.83	-0.76	-1.18	0.25	0.94	-1.56	-0.37	0.25	0.83
CD8+ perf+[3]	-0.12	-0.03	0.13	0.74	0.06	-0.22	0.13	0.99	-0.07	-0.08	0.13	0.91
NK cells	-0.44	-0.46	0.40	0.87	0.00	-0.91	0.40	0.83	-0.77	-0.12	0.40	0.95
NK perf+	0.00	-0.01	0.06	0.94	0.01	-0.02	0.06	0.88	0.01	-0.01	0.06	0.95
CD4+ T cells	0.80	0.95	0.54	0.84	1.61	0.13	0.54	0.68	-0.54	2.31	0.54	0.87
CD4+ perf+	-0.01	-0.36	0.12	0.99	-0.34	-0.04	0.12	0.89	-0.41	0.04	0.12	0.68
δγ+ T cells	6.38	7.53	1.00	0.98	7.04	6.88	1.00	0.99	9.67	4.21	1.00	0.78
δγ+ perf+	0.00	0.05	0.02	0.83	0.04	0.01	0.02	0.97	0.00	0.05	0.02	0.85
Monocytes	1.16	3.02	0.92	0.93	3.42	0.75	0.92	0.87	2.13	2.08	0.92	0.92
B cells	-0.27	0.01	0.20	0.89	-0.19	-0.05	0.20	0.92	-0.01	-0.23	0.20	0.83

[1]% = proportion relative to lymphocytes; [2]SEM = standard error of the means; [3]Perf+ = CD8+ T cells, NK cells, CD4+ and δγ+ T cells were stimulated with perforin to examine the functionality of these cells and how their functionality was affected by the different treatments.
