# Supplementary material for: Effects of pre-transport diet, transport duration and transport condition on immune cell subsets, haptoglobin, cortisol and bilirubin in young veal calves
Source: PLoS One. 2021 Feb 16;16(2):e0246959. doi: 10.1371/journal.pone.0246959 (PMC7886138; doi:10.1371/journal.pone.0246959)
Supplement: Attachment — Submitted filename: Response to reviewers.docx [file pone.0246959.s004.docx]

**RESPONSE TO REVIEWERS**

Dear Sir/Madam,

Thank you for reserving time to judge the paper and for your feedback. Please find below the list of changes we have made in the manuscript according to your comments. Our answers are indicated as **Authors.**

**Journal requirements**

1. Please ensure that your manuscript meets PLOS ONE's style requirements, including those for file naming. The PLOS ONE style templates can be found at

<https://journals.plos.org/plosone/s/file?id=wjVg/PLOSOne_formatting_sample_main_body.pdf> and

<https://journals.plos.org/plosone/s/file?id=ba62/PLOSOne_formatting_sample_title_authors_affiliations.pdf>

**Authors:** We have checked the manuscript and it meets PLOS ONE's style requirements. We have made few corrections in L 277- 279 in the new manuscript.

2. In your Methods section, please state the volume of the blood samples collected for use in your study.

**Authors:** We have added the volume of the blood samples collected during the experiment (see L148 in the new manuscript).

3. In your Methods section, please include a comment about the state of the animals following this research. Were they euthanized or housed for use in further research? If any animals were sacrificed by the authors, please include the method of euthanasia and describe any efforts that were undertaken to reduce animal suffering.

**Authors:** The animals included in the current research followed the normal production cycle of the veal industry, at the end of which calves were slaughtered and their meat was used for human consumption (see L136-138 in the new manuscript).

**Reviewer 1**

1. Is the manuscript technically sound, and do the data support the conclusions?

The manuscript must describe a technically sound piece of scientific research with data that supports the conclusions. Experiments must have been conducted rigorously, with appropriate controls, replication, and sample sizes. The conclusions must be drawn appropriately based on the data presented.

Reviewer #1: Yes

2. Has the statistical analysis been performed appropriately and rigorously?

Reviewer #1: Yes

3. Have the authors made all data underlying the findings in their manuscript fully available?

The [PLOS Data policy](https://eur03.safelinks.protection.outlook.com/?url=http%3A%2F%2Fwww.plosone.org%2Fstatic%2Fpolicies.action%23sharing&data=04%7C01%7C%7C0d8e5af16df346670ea208d89aadd4c5%7C27d137e5761f4dc1af88d26430abb18f%7C0%7C1%7C637429419093073783%7CUnknown%7CTWFpbGZsb3d8eyJWIjoiMC4wLjAwMDAiLCJQIjoiV2luMzIiLCJBTiI6Ik1haWwiLCJXVCI6Mn0%3D%7C1000&sdata=LypY1ejzyIMZLst8xT04BMhXS3LLpMKozZw3dbFGrhw%3D&reserved=0) requires authors to make all data underlying the findings described in their manuscript fully available without restriction, with rare exception (please refer to the Data Availability Statement in the manuscript PDF file). The data should be provided as part of the manuscript or its supporting information, or deposited to a public repository. For example, in addition to summary statistics, the data points behind means, medians and variance measures should be available. If there are restrictions on publicly sharing data—e.g. participant privacy or use of data from a third party—those must be specified.

Reviewer #1: Yes

**Authors:** We would like to provide a new version of the Data Availability Statement (please check the cover letter).

4. Is the manuscript presented in an intelligible fashion and written in standard English?

PLOS ONE does not copyedit accepted manuscripts, so the language in submitted articles must be clear, correct, and unambiguous. Any typographical or grammatical errors should be corrected at revision, so please note any specific errors here.

 Reviewer #1: No

**Authors:** We have checked the manuscript and made all corrections raised by the reviewer.

5. Review Comments to the Author

Please use the space provided to explain your answers to the questions above. You may also include additional comments for the author, including concerns about dual publication, research ethics, or publication ethics. (Please upload your review as an attachment if it exceeds 20,000 characters)

Reviewer #1: The aim of this study was to investigate effects of pre-transport diets, transport durations and transport conditions on immune cell subsets, haptoglobin, cortisol and bilirubin of young calves upon arrival at the veal farm. This reviewer considers that the manuscript is suitable for publication. Materials and methods were extensively described, specially the statistical analysis and results were properly discussed. I only have some grammar corrections as follows:

Line 28: Please, separate the word fowcytometry

**Authors:** we have corrected the word in the manuscript (see L28 in the new manuscript).

Lines 85 – 89: please, compile these two sentences in one sentence.

**Authors:** we have combined the two sentences into one (see L 85-88 in the new manuscript).

Line 146, 235, 268, 401, 412, 449, 486 and 591: please, change centre for center.

**Authors:** we have corrected the word in the manuscript (see L146, 235, 268, 401, 412, 449, 486 and 591 in the new manuscript).

Line 162: Americans spell it "titer" while the English spell it "titre".

**Authors:** we have corrected the word in the manuscript (see L162 in the new manuscript).

Line 194: Please change “permeabilized with a permeabilization” for “permeabilised with a permeabilisation”.

**Authors:** we have corrected the words in the manuscript (see L194 in the new manuscript).

Line 199: Same as above

**Authors:** we have corrected the word in the manuscript (see L199 in the new manuscript).

Table 4, 5 and 6 title: please, change centre for center.

**Authors:** we have corrected the word in tables 4, 5, 6 (see L347, 351 and 380 in the new manuscript).

Line 463: Please, change “However, there are to our knowledge no studies” to “However, to our knowledge, there are no studies…”

**Authors:** we have corrected the sentence (see L463-464 in the new manuscript).

Line 576: Please, change hypothesized for hypothesized

**Authors:** we have corrected the word (see L576 in the new manuscript).

Definition and clarity of main images must be improved.

**Authors:** we have improved the quality, definition and clarity of the figures (please see new attached files).
